# The Road to Elimination: Current State of Schistosomiasis Research and Progress Towards the End Game

**DOI:** 10.3389/fimmu.2022.846108

**Published:** 2022-05-03

**Authors:** Paul Ogongo, Ruth K. Nyakundi, Gerald K. Chege, Lucy Ochola

**Affiliations:** ^1^ Division of Experimental Medicine, Department of Medicine, University of California, San Francisco, San Francisco, CA, United States; ^2^ Department of Tropical and Infectious Diseases, Institute of Primate Research, Nairobi, Kenya; ^3^ Primate Unit & Delft Animal Centre, South African Medical Research Council, Cape Town, South Africa; ^4^ Department of Pathology, University of Cape Town, Cape Town, South Africa; ^5^ Department of Environmental Health, School of Behavioural and Lifestyle Sciences, Faculty of Health Sciences, Nelson Mandela University, Gqeberha, South Africa

**Keywords:** schistosomiasis, diagnosis, vaccines, elimination, research

## Abstract

The new WHO Roadmap for Neglected Tropical Diseases targets the global elimination of schistosomiasis as a public health problem. To date, control strategies have focused on effective diagnostics, mass drug administration, complementary and integrative public health interventions. Non-mammalian intermediate hosts and other vertebrates promote transmission of schistosomiasis and have been utilized as experimental model systems. Experimental animal models that recapitulate schistosomiasis immunology, disease progression, and pathology observed in humans are important in testing and validation of control interventions. We discuss the pivotal value of these models in contributing to elimination of schistosomiasis. Treatment of schistosomiasis relies heavily on mass drug administration of praziquantel whose efficacy is comprised due to re-infections and experimental systems have revealed the inability to kill juvenile schistosomes. In terms of diagnosis, nonhuman primate models have demonstrated the low sensitivity of the gold standard Kato Katz smear technique. Antibody assays are valuable tools for evaluating efficacy of candidate vaccines, and sera from graded infection experiments are useful for evaluating diagnostic sensitivity of different targets. Lastly, the presence of Schistosomes can compromise the efficacy of vaccines to other infectious diseases and its elimination will benefit control programs of the other diseases. As the focus moves towards schistosomiasis elimination, it will be critical to integrate treatment, diagnostics, novel research tools such as sequencing, improved understanding of disease pathogenesis and utilization of experimental models to assist with evaluating performance of new approaches.

## Introduction

The World Health Organization (WHO) has renewed its efforts in the fight against Neglected Tropical Diseases (NTDs) by recently revising its road-map for NTDs 2021-2030 to achieve sustainable development goals (SDGs) (1). This roadmap was developed through an extensive global consultation with NTD stakeholders, with endorsement by 194 member states at the 73rd World health assembly in November 2020 (2). The overall cross cutting activities that would aide in further reducing the burden of NTDs are highlighted in **Table 1** and these fall into three pillars namely (i) action to reduce incidence, prevalence, morbidity, disability and death (ii) Cross-cutting approaches using integrated delivery of interventions for NTDs and (iii) Change operating models so that countries own NTD programs. In addition to the three pillars, it’s important to note that interventions that lead to a decline in the transmission of NTDs may also have an indirect benefit on the control of other tropical diseases such as tuberculosis (TB) through potentially improved performance of childhood vaccines. To date, the overall reduction in health, social and economic burden of NTDs is attributed to multiple integrated programs that include mass drug administration, single/combination drugs, water hygiene and sanitation (1).

**Table 1:** Cross-cutting activities for WHO roadmap 2021-2030.

	Cross cutting issues
Pillar 1	Actions to reduce incidence, prevalence, morbidity, disability and death • Scientific advances • Fill knowledge gap • New interventions • Effective, standardized and affordable diagnostics
Pillar 2	Cross-cutting approaches using integrated delivery of interventions for NTDs • Mainstream with national health systems under universal health coverage • Enhance coordination among stakeholders and related programs (WASH, vector control.
Pillar 3	Change operating models so country own NTD programs • Collection and reporting of data on NTDs disaggregated by gender

The neglected tropical disease of schistosomiasis caused by blood flukes of the genus *Schistosoma* continues to be a scourge to humankind. It afflicts 252million people in the tropics and sub-tropics (3) causing approximately 70 million Disability Adjusted Life Years (DALYs). It is the third most devastating tropical disease globally and is a major cause of morbidity and mortality in Africa, South America, the Caribbean, Middle East and Asia. More than 78 countries are affected and nearly 800 million people are exposed to the diseases. Efforts to control NTDs include a multidisciplinary and integrated approach of understanding the environment, mode of transmission, immunology of the disease, ensuring improved access to diagnosis, treatment, and vaccine development. For schistosomiasis, a lot of efforts have focused on mass drug administration (MDA), improving access to water sanitation and hygiene (WASH). This has reduced intensity of infection, however re-infection still remains a challenge (4, 5). Therefore, for us to achieve total cure and elimination, additional interventions are required.

In this paper we review the current actions taken to reduce the incidence, prevalence, morbidity, disability and death that focus on Pillar 1 of WHO’s roadmap. The paper will highlight the following: Schistosomiasis immunology, up-to-date status of diagnostics, treatment and vaccination strategies and provide, in context, how the baboon model can be used to test and validate novel tools to influence effectiveness of national health systems and programs and in turn elimination of schistosomiasis.

## Immunology of Schistosomiasis

Schistosomes are transmitted by trematode snails that infect its host during contact with infested water (6). The two predominant species of *Schistosoma* infecting man are *S. mansoni* and *S. haematobium* while, *S. japonicum* is zoonotic infecting man as well as wild and domestic animals. *S. mansoni* is transmitted by *Biomphalaria* snails and *S. japonicum* by *Oncomelania* causes intestinal schistosomiasis and in humans the mature adults inhabit the inferior mesenteric veins and tributaries laying eggs with a lateral spine and small vestigial spine respectively (7). While *S. haematobium* transmitted by the genus *Bulinus* causes urinary or vesical schistosomiasis with adults inhabiting mainly veins of the vesical plexus and lay eggs with a terminal spine. The parasite has several stages, penetrating cercariae in the skin that transform into schistosomula which enter into the vasculature and travel *via* the pulmonary artery to the lungs (8). They then exit the lungs and re-enter the venous circulation and migrate to the perivesicular veins (*S. haematobium*) or mesenteric venules (*S. mansoni, S. japonicum*) and mature into adult male and female that pair up and are impervious to immune attack. However, protective immunity against schistosomula may develop over time once parasites begin to die either naturally or through treatment with antihelminthic drugs.

The acute phase of the disease develops 4-8 weeks post infection (9) and individuals present with fever and abdominal pain (10). Adult worms can survive for long durations of three to ten years (11, 12) and lay hundreds to thousands of eggs (6). These either leave the body *via* stool (*S. mansoni, S. japonicum*) or urine (*S. haematobium*) or become trapped in the liver causing the primary etiology of shistosomiasis (13). Eggs are continuously deposited causing a granulomatous response resulting over time in chronic inflammation, with fibrosis and eventually severe organ damage (14, 15). In the case of *S. haematobium*, ureteral obstruction, squamous bladder cancer, genital lesions are observed while *S. mansoni* causes periportal fibrosis with portal hypertension (13). *S. mansoni* infection in murine models have shown that most pathology is triggered by CD4+ Th2 driven granulomatous response against schistosome eggs and the antigens they secrete, reviewed in (16). Granuloma development and fibrosis formation are similar in both *S. mansoni* and *S. japonicum*, with the latter inducing a severe granulomatous response that is neutrophilic (17). In mice, Warren et al. (18) observed that the immune response to purified soluble egg antigen (SEA) is of a delayed type hypersensitivity while in *S. japonicum* it’s an immediate type hypersensitivity. Granuloma formation primarily driven by schistosome eggs induces IL-1β secretion from immune cells which activates the inflammasome pathway (19, 20) in both bone marrow derived dendritic cells (BMDCs) as well as \ *in vivo* (21). The inflammasome network engaged by schistosome eggs involves both immune and non-immune cells, reviewed extensively in (22). In general, the key immune cells in granulomas consist of lymphocytes, macrophages and eosinophils that contain egg proteolytic enzymes that prevent tissue damage. Egg-induced granuloma lead to chronic schistosomiasis.

T lymphocytes play an important role in regulating the pathological immune response to schistosomiasis. Studies in mice have shown that granuloma formation leads to a shift from Th1 to Th2 response. Where, IFNγ dominates the Th1 response, whilst presence of granulomas correlates with increased production of egg antigen specific tumor necrosis factor (TNF-α), interleukin-4 (IL-4), IL-5, IL-13 (23–29). These cytokines stimulate the B cells to proliferate to plasma cells which produce antibodies especially IgE. Down modulation is in part mediated by IL-10 and parasite antigen specific antibodies (29–31). Recent studies in mice have further pointed to the role of IL-17 in *S. mansoni* induced pathology with high levels correlating with severe liver pathology (32–34). Similar observations have been corroborated in humans (35, 36). Another class of T cells, T follicular helper cells (Tfh) that provide B cells with help (37) were shown to differentiate through induction of *S. mansoni* eggs. In mice infected with *S. japonicum* Tfh cells promote liver granulomas and fibrogenesis (38, 39). In clinical studies, Tfh cells were associated with immune responses to both acute and chronic human schistosomiasis (40, 41) and IL-4 producing Tfh cells are thought to provide acquired resistance to schistosome re-infection (42).

In the host, all parasite stages are able to stimulate the immune system *via* antigenic moieties (43–45) which result in strong humoral and cellular responses with these responses increasing during chronic stage of disease. Every process from initial exposure to cercariae through Th1 to Th2 cell differentiation to fibrogenesis in the liver, are tightly regulated by constant recalibration of the immune homeostasis (46). These processes are maintained by regulatory CD4+ T cells (Tregs) that inhibit T cell proliferation and mediate the magnitude of immunity to invading pathogens (47). Tregs suppress dendritic cells, activate and mediate Th2 responses, inhibit granuloma development and fibrosis during schistosome infection. Natural Tregs express a transcription factor Forkhead box protein 3 (Foxp3) and this has allowed investigators to separate natural T regs from inducible Tregs (48, 49). Singh and others, 2005 (50), observed a significant increase in the percentage of granuloma natural Tregs (CD4+CD25+Foxp3+) at 8 to 16 weeks post infection in mice. As the disease progressed to the chronic stage of egg induced inflammation, the nTregs phenotype changed to CD103-expressing nTregs. This is thought to support immunosuppression (51).

## Schistosomiasis in Experimental Model Systems

Studies from natural populations and Olive baboons experimentally infected with *S. mansoni* found that majority of animals had a high serum concentration of parasite specific IgG antibodies, with 68% positive for soluble worm antigen (SWAP)-specific IgG antibodies, while 54% were positive for soluble egg antigen (SEA)-specific IgG antibodies. While less than one third of animals were positive for SWAP-specific and SEA specific IgM antibodies (52–54). Olive baboons further develop granulomas from week 6 post infection and schistosome egg antigen induces production of IL-2, IL-4, Il-5, TGFB and IL-10 and these decline during the chronic stage. These cytokines further activate eosinophils and mast cells (55). The Th-2 immune environment serves to protect the host against severe egg induced morbidity. In nature, transmission of schistosomiasis further involves non mammalian hosts (56) and small mammals (rodents) (57). Murine models, though effective, develop resistance to new infections (58), while in Olive baboons the disease progresses in the same manner as humans (reviewed by (59). Baboons also exhibit resistance to reinfection after treatment with praziquantel similar to humans (52, 60). These animals have been exploited as experimental model systems for understanding schistosomiasis disease pathogenesis, developing and testing of drugs, diagnostics and vaccines.

## Schistosomiasis Control Strategies

Schistosomiasis control programs have focused on a number of strategies that include environmental control, diagnosis and chemotherapy, improved health education, provision of clean water, sanitation and disease surveillance. Elimination of the vector snails *via* mollusciciding, environmental modification, self-protection and use of chemical repellent or niclosamide impregnated clothes, safe water supply, construction of latrines have all served to control the disease (61). However, given the changing climatic conditions environmental control remains an arduous task (62). Diagnosis and chemotherapy have played an important role in control of schistosomiasis. First introduced in the 1970s, praziquantel (PZQ) remains the drug of choice for treating schistosomiasis and the key public health strategy to combat schistosomiasis (63, 64). With recent efforts based on mass drug administration (MDA) of PZQ as preventive chemotherapy in *Schistosoma* endemic regions focused on school going children and other risk groups (65). Overall, chemotherapy facilitated morbidity control and contributed to decrease in schistosomiasis cases (66–69). PZQ is administered at a dose of 40mg/kg given annually. However, gains made have been hampered by re-infections in humans. PZQ is only effective against adult worms of all schistosome species infecting humans and is safe, however it is unable to eliminate juvenile worms (immature parasites) or migrating schistosomulae (70). Studies in *S. mansoni* infected experimental mammals further showed that PZQ is unable to kill juvenile schistosomes 28 days post infection (71, 72). This has implications on control programs where reduced susceptibility to PZQ has been observed especially in areas of intense transmission (70), with studies in Egypt observing emergence of schistosomes from eggs from patients treated with PZQ (73) and low cure rates in Senegal (74). There is further evidence to show that MDA may reduce population immunity in the long term and if stopped can lead to large rebounds in egg counts from *S. haematobium* infections (5, 75). Though MDA remains an invaluable tool in the control and elimination of schistosomiasis, the dark side of these interventions is the potential onset of drug resistance to antihelmintic drugs (PZQ) that could in turn jeopardize all global efforts and re-infection in the community (76, 77). Attempts have been made to evaluate higher doses of PZQ (>60mg/kg) and a recent pharmacokinetic study recommended a higher dosage to achieve therapeutic cure in young children (78). Until alternatives are discovered to PZQ it will still remain valuable in treating schistosomiasis.

A large proportion of individuals in schistosomiasis endemic regions live in environments with inadequate access to water and sanitation, infrastructure and unsafe hygiene practices which results in constant risk of infections from parasites. A number of countries in South America, Asia and Africa have adopted WASH practices as part of their national NTD mitigation strategy (79, 80). In countries where WASH was introduced and where people had access to adequate sanitation at home statistically significant lower odds of infection with schistosomiasis were observed (79, 80). In Tanzania, communities engaged in WASH campaigns that included safe excreta disposal, improved availability and use of safe drinking water and handwashing practices (81), this resulted in control of schistosomiasis outbreak in Ngorongoro district, however, they noted the requirement of sustained government involvement to ensure access to reliable WASH programs. Overall, integrating all these interventions leads to a decline in schistosomiasis transmission as observed in Oman (82).

## Diagnosis of Schistosoma Species Infections

The WHO appreciates that a multisectoral action is necessary for the control of schistosomiasis and highlights diagnostics, together with monitoring and evaluation, access and logistics, advocacy and funding as pivotal in realizing the 2030 targets (1, 2). Schistosomiasis is targeted for elimination as a public health problem, defined as <1% of heavy intensity infections with improvement in diagnostic approaches identified as a critical requirement to reach this target. It will require a deliberate investment and focus into different facets of diagnostics research because specific areas require diagnostic tools with different capabilities. For example, large epidemiological mapping will require standardized, sensitive point-of-care diagnostic for use in various prevalence settings; creation of a repository of sera, urine and stools for development, validation and evaluation of new diagnostic targets; development of test for resistance to praziquantel treatment especially in areas with high disease transmission; develop point-of-care diagnostic for genital manifestations particularly tests that are not invasive like biopsy sampling; and develop molecular test for xenomonitoring and surveillance which has been effective in other NTDs like Onchocerciasis. Here we discuss the available methods for diagnosis of *schistosomiasis*, highlight the advantages and limitations of each method (**Table 2**), and possible improvements, and finally highlight the opportunities that exist for discovery of new targets of parasite origin with diagnostic potential.

**Table 2:** Performance characteristics of current schistosome diagnostic methods.

Technique	Advantages	Limitations
**Parasitology based diagnostics**		
Kato-Katz smear (36)	• Simple technology. • Affordable. • Rapid for estimation of infection burden and MDA success in high transmission areas. • Egg burden correlates with infection intensity.	• Poor sensitivity and unsuitable for use in low infection intensity settings • Performance is influenced by changes in fecal fiber content. • Affected by day-to-day variation in egg excretion. • Sensitivity cannot be improved below 20 epg • Exposes the user to potential infectious materials in the sample.
Concentration methods@ (41, 52–55, 57)	• Can be used on fixed and stored stool samples • Can accommodate a large amount of starting sample • Circumvents the problems associated with consistency of the fecal material	• Takes a long time to perform. • Generally superior to Kato- Katz smear, the ‘gold standard’ • Some methods like FLOTAC can distort the appearance of eggs increasing chances of false negative results.
**Antibody based diagnosis** (62, 63, 65, 66, 68, 83, 84)		
	• Applicable early after exposure to infection (the prepatent phase). • Suitable for use in areas of low transmission. • Generally more sensitive than parasitology based methods. • Performance (sensitivity and specificity) can be improved by using recombinant proteins as target antigens. • Ideal for monitoring vaccine induced responses. • The performance can be improved depending on the platform, e.g immunoblot is sensitive than ELISA. • Can be modified to point of care diagnostic rapid diagnostic kits.	• Antibody responses to crude antigens can cross-react with other helminths. • Cannot distinguish past from ongoing infections, thus unsuitable for monitoring MDA success. • ELISA based assays require instrumentation that depend on electricity. • Sensitivity threshold is moderate in the prepatent period. • Antibody levels cannot be used as a surrogate for infection intensity. • Takes a long time to perform.
**Molecular based diagnosis**		
PCR techniques (33, 42, 85–88)	• Have high and sensitivity and suitable for low infection burden. • Not affected by cross-reactivity because primers are specific for schistosomes. • PCR Ct values may be used as surrogates for infection intensity. • Performance can be improved depending on technique.	• Detection of cell-free DNA cannot distinguish active from previous infections. • Require expensive machines to run and skilled training. • Takes a long time to perform. • The primers are temperature sensitive and require refrigeration.
LAMP (83, 89–91)	• Ideal for xenomonitoring studies. • Have a high specificity and sensitivity. • Technique reactions can be performed in a water bath.	• The primers are temperature sensitive and require refrigeration.
**Circulating glycan-based method** (84, 92–96)		
	• Demonstrates the presence of active infection. • Ideal for monitoring success of MDA programs. • Can be performed on urine, removing the need for invasive sampling. • Available as point-of-care rapid test-kit. • Suitable for low transmission settings. • Specific for *Schistosoma* genus.	• Not ideal for prepatent phase of infection. • Improvement of sensitivity requires concentration of samples that can be costly.

MDA, Mass drug administration; epg , eggs per gram of fecal sample; @, combination of different techniques including FLOTAC; Percoll concentration; Helmintex; saline gradient; PCR, polymerase chain reaction; LAMP , loop mediated Isothermal amplification.

### Parasitology Based Diagnostics

The demonstration of eggs in stool (*S. mansoni, S. japonicum* and other less common intestinal forms) or urine (*S. haematobium*) is regarded in clinical settings as the definitive confirmation of infection with schistosomes, and as such, is ‘the gold standard’ reference in most studies (85, 97, 98). Conversely, the absence of eggs is regarded as a measure of success of drug administration (86) or absence of community transmission. Before MDA in schistosomiasis control campaigns, parasitological methods, or the use of questionnaires for self-reporting of characteristic symptoms (87, 99) are frequently used to identify target populations.

The most widely used parasitological method is the Kato-Katz technique because it requires relatively simple technology, based on a slide template that can take 50mg of fecal material, allowing a rapid estimation of infection burden, expressed as eggs per gram (epg) of a stool sample (100). For urine microscopy, detection of eggs is preceded by urine filtration technique using nylon, paper or polycarbonate filters to concentrate the eggs which are expressed as eggs per 10ml of urine (88). One limitation of Kato-Katz smears is that the sensitivity is influenced by the consistency of the fecal material (101, 102); for example, moderate to heavy infections may cause diarrhoea and the availability of fruit can lead to changes in fecal fibre content (89), both of which can mask egg identification. The results can also be affected by day-to-day variability in egg excretion (83, 90, 101) which determines the distribution of eggs in the sample. The biggest drawback of the technique is that it has an automatic detection limit of 20 epg, based on the ∼50mg capacity of the chamber (100). A cross-sectional study identified *S. haematobium* eggs in semen of fishermen, demonstrating the lodgment of eggs in the reproductive system with the implication that quantification of infection burden using urine alone may underestimate infections (91). Although the positivity rate of Kato-Katz is directly proportional to the number of slides and fecal samples examined (103, 104), it is logistically challenging in large epidemiological studies to examine multiple replicate slides. Given that the WHO recommends that stool sampling be done on three consecutive days to have a reliable analysis brings to the fore how challenging this is in resource limited settings (105).

Our studies in the baboon model of schistosomiasis (92–95, 106, 107) also reviewed in (96, 108) confirmed the low sensitivity of Kato-Katz smear. Since it is possible to recover adult worms from infected animals by portal perfusion, we compared epg of faeces as a surrogate estimate of worm burden and determined a detection threshold of 40 worms (107). There were no eggs detected in nine replicate smears from three animals with a measurable worm burden (25 or more worms) which would have been false negatives by the Kato-Katz smear technique. On a positive note, however, the R^2^ of 0.72 shows that fecal egg counts are good quantitative surrogate estimates of actual worm burden. When adjusted for sex-ratio (106), this threshold is equivalent to approximately 16 worm pairs.

Given the small amount of sample (∼50mg capacity) for the Kato-Katz smear chamber, there have been improvements to circumvent low frequency of eggs in stool, especially in low burden infections, and their random distribution to enrich for eggs prior to counting using larger amounts of stool samples. One of the advantages of these methods is that some can be used on fixed and stored fecal samples allowing for flexibility to transport samples from the field and run later. FLOTAC method (109) and ether concentration (110) both estimated a higher % prevalence of *S. mansoni* infections in human fecal samples compared to triplicate Kato-Katz smears. However, FLOTAC method produced lower quantitative epg counts implying that many eggs were not recovered by floatation and the fragility of schistosome egg in the FLOTAC zinc sulphate solution was confirmed in images (111). To get around the problem of egg fragility, a biocompatible 60% Percoll solution in 0.9% saline has been used as a separation medium (84). In rhesus macaques, chimpanzees, baboons and humans the method has consistently shown superiority to conventional Kato-Katz smear (111; our unpublished data). However, application of the Percoll method is still limited to research settings rather than MDA programs as it requires viable eggs, a centrifuge, and an expensive reagent.

Helmintex technique is also based on the physical properties of eggs. It starts with 30 gm of fecal material, processed by passage through a series of sieves, excluding larger debris but allowing colloidal particles to travel in the flow-through (112). The eggs are then stained and concentrated further using paramagnetic beads, before counting (113). Helmintex proved significantly superior to both the Kato-Katz smear and a saline gradient method in diagnosing individuals with a light infection (114). The main hindrance of this method is the time taken to process each sample that would be unsuitable for monitoring effectiveness of programs like the MDA.

Parasitology based methods are suitable for use in areas of high and medium infection intensity, but their main drawback is the relatively poor sensitivity, particularly the most preferred Kato-Katz smears. Because parasitological methods are designed to detect eggs, they are also unable to diagnose recent infections where worms have not yet started to produce eggs (the prepatent period) (115). As a means of monitoring success of control programs, it is important to note that following treatment, the number of negative stool tests increases, and the performance characteristics of standard diagnostic tests decrease with regards to sensitivity and negative predictive value and test-to-test variability increases (116). Additionally, the sensitivity of parasitological methods diminishes when prevalence and intensity of infection are low, making them less appropriate for low-endemic areas or post-treatment situations.

### Antibody Based Diagnosis

Due to poor sensitivity of parasitological tests and the fact that it takes approximately 40 days post infection before beginning of egg excretion (94, 117), alternative diagnostic approaches like antibody-based assays have been developed (118), mostly designed as ELISA platform. These tests are, in general, well suited for areas with low transmission and can be rapid tests for travelers returning from endemic areas (119, 120). Most antibody assays report on immunoglobulin G (IgG), and rarely immunoglobulin M (IgM) (121, 122), that target crude antigen extracts like cercarial secretions, schistosome egg antigen (SEA), soluble adult worm antigen preparation (SWAP), or can be constructed to detect purified or recombinant antigens (106, 123, 124). Soon after exposure, patients present with an acute inflammatory response driven by the body’s immune response to schistosomula migration (125) and a positive antibody test is commonly the earliest diagnostic laboratory result. Still, a large fraction of patients will initially test negative (126, 127), meaning timely treatment is not provided for false negative cases. The negative result could be explained, at least in part, by the choice of antigen used in the assay.

While antibody-based assays are more sensitive than Kato-Katz smears, especially in low infection intensity, these assays have key limitations. First, and most important, parasite-specific antibodies remain detectable in the circulation for years after the infection has been cleared (108, 128). This implies that antibody-based assays are not suitable for monitoring success of MDA programs especially where the target antigen is crude parasite extract. As a result, anti-schistosome antibody titers in serum fail to distinguish between current and previous infections. Secondly, antibody levels in serum do not necessarily correlate with the intensity of infection as determined by mean epg of faeces (115). Thirdly, because of shared antigenic epitopes, cross-reactivity with other helminthic infections is a challenge since schistosomiasis and soil transmitted helminth coinfections is a norm rather than an exception in most settings. The performance of antibody-based assays in terms of sensitivity and specificity can be improved upon by selecting specific antigens for coating ELISA plates (121, 123, 129). However, little progress has been made in terms of developing single specific targets as diagnostics (130) despite sufficient information known about the secreted proteins from the gut and tegument of adult worms, and from mature eggs [reviewed in (130)].

Antibody-based assays are useful in monitoring vaccine induced responses in several infections. In the baboon model, our vaccination studies demonstrated that levels of schistosome specific IgG after vaccination with radiation attenuated cercariae correlated best with protection (92), and IgG levels specific to Schistosoma antioxidant enzymes, Cu-Zn superoxide dismutase (SmCTSOD) and glutathione S peroxidase (SmGPX), were stimulated after immunization and remained elevated 10 weeks post challenge compared to control unvaccinated animals (131). Thus, antibody assays are important for evaluating vaccine responses to candidate vaccines in development.

Serological techniques can be highly sensitive depending on the platform used. For example, the development of an immunoblotting assay using *S. mansoni* adult worm extracts improved the performances of serological screening and proved to be relevant in intestinal schistosomiasis (132). However, a typical ELISA technique requires considerable time before results are generated and relies on instrumentation that may not be available in all settings endemic for schistosomiasis. Therefore, antibody-based assays need to be designed into portable point-of-care rapid test kits that do not require cold-chain storage to meet the requirements of field studies for disease prevalence screening. The readout of such rapid test kits should be easy to interpret and preferably compatible with new technologies, for example the ability to capture the results with mobile phone cameras that can be presented to public health personnel for treatment initiation.

### Molecular Techniques

Polymerase chain reaction (PCR)-based assays use different targets to detect *Schistosoma* DNA, including the ribosomal subunits 18s rDNA, 28s rDNA and SSU-rRNA (133, 134), mitochondrial genes (nicotinamide adenine dinucleotide hydrogen – NADH-I, NADH-3), internal transcriber-spacer-2 sequence – ITS2), and the cytochrome c oxidase-COX I (135). The most studied target is the 121 bp tandem repeat (103, 136–138), across different species of schistosomes because of the high copy number (600, 000 per schistosome cell) making for high detection sensitivity even if only a few DNA fragments are present in the sample. Apart from whole eggs in stool, urine and tissue biopsies, schistosome DNA can also be amplified from cell-free parasite DNA (cfDNA) released from schistosome stages (schistosomula, adult worms and eggs), which could derive from dead or decaying parasites within the circulation and tissues, active shedding from the parasite or from disintegrating inactive eggs (139–141). cfDNA can be detected in serum, plasma and bio-fluids such as urine, saliva and cerebrospinal fluid (86, 140, 142, 143). It has been demonstrated that cfDNA can be readily detected in active, low-intensity infections and low prevalence schistosomiasis areas, using Droplet Digital PCR (ddPCR) and rt-PCR (141, 143). While the detection of cfDNA released from different stages of schsitosomes development by the PCR-based assays can enable the diagnosis of the infection before the beginning of egg excretion, cfDNA may not be ideal for monitoring prevalence soon after treatment. Indeed, cfDNA has been detected in semen several weeks after a single dose of praziquantel (144).

Several studies have compared sensitivity of PCR-based assays with other diagnostic methods, and in nearly all cases, the PCR-assays proved superior to the other methods. Real-time PCR (rt-PCR) showed a higher sensitivity in comparison to parasitology and serology-based assay when treatment success was assessed in a small study of children presenting with symptoms of acute schistosomiasis. The positivity rate for parasitological, serology and rt-PCR were 3/7 (44.9%), 4/7 (57.1%) and 6/7 (85.7%), respectively, while urinary antigen was detected in all infected children before treatment (117). At 22 months post treatment, all the children were negative for schistosomiasis by all the tests except 1 who was positive by rt-PCR. DNA late positivity in egg-negative individuals may occur post-PZQ administration (145), and could suggest an ongoing active infection, resulting from continuous DNA release from tissue trapped eggs or single gender-induced infection. In our baboon model studies, we observed that in some animals more male worms were recovered after portal perfusion than female worms (131).

In comparison to microscopy, PCR significantly increased the sensitivity of diagnosis of *S. mansoni* in stools from 33.7% to 48.8%, and serum PCR positivity rates slowly declined from 93.8% at day 30 to 8.3% at day 360 post treatment while antibody detection remained positive after 1 year (128). In this study, agreement between results of microscopy and PCR varied depending on the type of sample demonstrating the relevance of the starting material for the outcome of molecular based tests. Of note, PCR Ct values appeared to correlate with microscopic results as median Ct values of PCR assay were significantly lower in samples with microscopic egg detection, compared to those with negative microscopic results (128). Since egg burden is a correlate of infection intensity (100, 107, 146), the observed inverse correlation between PCR Ct values and egg detection in this study and separately in (147) implies that PCR Ct values could also be used as a surrogate for infection intensity.

When a combination of three parasitological tests - 18 Kato-Katz slides, saline gradient and Helmintex techniques – was used as the consolidated reference standard (CRS), rt-PCR was positive in 117 out of 215 tested samples with 91.4% sensitivity, 80.2% specificity and good concordance with the CRS (kappa= 0.71) (147). Importantly, rt-PCR identified 86.9% of the individuals eliminating less than 12 epg of feces, demonstrating much better performance than POC-CCA^®^ at 50.8%. However, there were 9 individuals with eggs in any of the parasitological exams who showed no reactivity in the rt-PCR assay. This discordance can arise from the possibility that there were no schistosome eggs in the starting fecal material, or even inhibition of PCR amplification. On the other hand, rt-PCR showed a DNA sequence compatible with *S. mansoni* in 22 individuals that were considered egg negative in all the parasitological techniques.

The sensitivity of PCR-based assays has been improved using q-PCR technique to a limit of detection of 0.38fg (equivalent of less than a single cell) of *S. mansoni* DNA (103), which corresponds to approximately 0.00065 times its genome (133). Compared to Kato-Katz technique with two slides (used in Schistosome control programs in Brazil) as a definitive diagnostic, qPCR had 95.7% sensitivity, 81.6% specificity and 83.8% accuracy (103). When the number of Kato-Katz slides were increased to 24, qPCR presented 96.7% sensitivity, 87.2% specificity and 89.2% accuracy. Based on the results from the “reference test” (24 Kato-Katz slides + Saline Gradient method-SG) the qPCR presented 82.6%, 93.1%, and 89.8% of sensitivity, specificity, and accuracy rates, respectively. The positivity of qPCR was higher than that of the 24 Kato-Katz slides, SG techniques (103) or Kato-Katz and Spontaneous Sedimentation techniques combined (148) when tested in a low endemicity area. As the number of infection intensity reduces and the number of epg of feces becomes smaller (149), there is an increased likelihood of discrepant results between PCR-based assays and parasitological tests. In regions where schistosomiasis and soil transmitted helminths are co-endemic, the qPCR technique is especially valuable since it is possible to multiplex for the diagnosis of other intestinal parasites.

Loop-mediated isothermal amplification (LAMP), can be an appealing alternative to PCR for its improved sensitivity because the technique uses four or six primers for the amplification of a single target gene at a single temperature step from 63 to 65°C, maintained at 65°C for 60 min (150–152). LAMP-assays have been extensively explored in xenomonitoring studies with encouraging success (153, 154). While LAMP technique removed the prohibitive need for expensive thermocycler machines, large scale field applications will require that LAMP reaction mixtures are provided premixed, ready for use and storable under field-laboratory conditions (150).

Overall, the detection of *Schistosoma* DNA by PCR is a promising, highly sensitive, and specific tool that could improve and facilitate the diagnosis of schistosomiasis, particularly in non-endemic countries where the parasite burden is lower (155–158). As discussed, it is possible to amplify cell-free DNA and enable detection of schistosomes during the pre-patent phase. But this is a double-edged sword; it can lead to positive results when the parasite has been eliminated by chemotherapy. Additionally, when the starting material is stool sample, then PCR-based assay suffers the same fate as parasitological tests in that the stool must contain eggs. The biggest impediment to application of molecular based diagnostics in large epidemiological settings remains the infrastructure requirement that is limiting in most regions endemic for schistosomiasis.

### Circulating Schistosome Glycan-Based Antigens

As an alternative to testing for reactive antibodies in infected individuals, the detection of parasite-derived antigens in circulation or urine (142) has been explored. Different assays were developed and took off mainly from the early 1990s (159–161) with the leaders in the field being the group of van Dam, Deelder and colleagues from Leiden, The Netherlands (162). The assays depend on adult worms vomiting circulating anodic antigens (CAA) and circulating cathodic antigens (CCA) into the host bloodstream (162–164) and the detection of their immunologically reactive O-linked glycan residues. These assays require generation of monoclonal antibodies specific for the glycan epitopes (165, 166). To generate the glycans, serum or urine samples are precipitated of proteins by trichloroacetic acid (TCA), releasing the glycans from immune complexes and leaving them in the supernatant (167, 168). The detection of CAA and CCA demonstrates only active infections and is thus suitable for assessing the effect of treatment on worm burden, with CAA and CCA measured by ELISA demonstrating high specificity (169, 170). Since CAA and CCA can be detected in urine, it means that invasive methods of sample collection like whole blood draw for serum can be avoided (162, 168).

Despite being highly specific (126, 171), detection of circulating antigens by ELISA was not superior to detection of eggs by Kato-Katz smears in areas of low endemicity (126, 172, 173). Again, making use of our baboon vaccination experiments, we demonstrated that the threshold of detection of CAA and CCA ELISA was 24 and 47 worms respectively (107), and R^2^ values of 0.74 and 0.46, respectively, indicating that CAA was a better predictor of worm burden than CCA. Given the great potential of employing the detection of circulating antigens for large epidemiological studies, considerable effort was invested in refining the technique that resulted in the development and commercialization of two urine CCA assays (115). The first of these was employed in studies of children with sensitivities and specificities in the low 80th percentiles when compared with stool egg data (174). The second assay was the lateral flow cassette-based designed as a point-of-care CCA assay (POC-CCA) (115), the introduction of which had a great impact in schistosome diagnostics. Consequently, the POC-CCA has been widely used in mass treatment programs in several African countries, where it has outperformed the fecal smear as a diagnostic tool (175–178).

Additional improvement of these tests has been the combination with various quantitative lateral flow (LF)-based assays utilizing up-converting phosphor (UCP) reporters (168, 178–180). Importantly, the UCP-LF assay for CAA detection has been adapted to a dry reagent format that is stable at ambient temperature and worldwide shipping without the need for a cold chain (181). As a result, CAA detection has been deployed in many countries and shown to be a highly sensitive diagnostic biomarker for detection of active schistosomiasis as reported in several Schistosomiasis Consortium for Operational Research and Evaluation (SCORE) studies, extensively reviewed in (182). Using baboon sera from previous vaccination experiments and graded infections, we obtained an improved resolution of the relationship between CAA levels and worms, in animals with low worm burden but still short of the desired ability to detect a single worm pair (108, 168).

Detection of schistosome CAA and CCA has been a good addition to the tool kit for monitoring success of MDA programs in different settings, including new trials in pregnant women and children under the age of 2 years (183). Notably, the CAA test is a genus-specific detecting various *Schistosoma* species including the veterinarian ones (184–186) which eliminates the challenges of cross-reactivity that is common in antibody-based assays. Additional improvement of the performance of these assays for wide application in regions with low transmission settings will be ideal for monitoring the progress towards the ultimate goal of elimination of schistosomiasis (187). However, some POC-CCA results can be subjective to the reader and the interpretation of the ‘trace’ test can vary greatly (176). In addition, the fact that CAA and CCA are products of regurgitation from the gut of the adult worm suggests that these antigens are inappropriate for diagnosis soon after exposure. Related to this and in areas of high transmission, there will be a need to perform more than one test to increase the chances of identifying individuals in whom chemotherapy did not clear juvenile worms. Additionally, to achieve maximum sensitivity there is need to concentrate the CAA in the serum or urine sample using centrifugal filters (188) which may be limiting in some settings.

### Identification of New Diagnostics Targets Using Proteomics and Bioinformatics Approaches

The complete sequencing, assembly, and annotation of *S. mansoni* genome (189) building on the prior advances in large scale sequencing of schistosome cDNAs (44) greatly opened the landscape of schistosomiasis research. Together with progresses in mass spectrometry, it became possible to determine, with improved precision, the protein composition of parasite fractions (190). Several research groups became actively involved in characterizing parasite proteins including Wilson and colleagues from the University of York, UK. This group characterized and described proteins from the different schistosome life cycle stages including invading cercariae (191), migrating schistosomula (192), proteins exposed at, or released from the adult tegument surface (193, 194), those vomited into the bloodstream from the parasite gut (195), and the products of the live mature eggs (196). This catalogue of schistosome proteins across all lifecycle stages, extensively reviewed in (108, 197), is resourceful for targeted choice of parasite proteins secreted into the host circulation and potentially in urine as well. Detection of antibodies against these proteins is appealing because some of the proteins, especially from the gut, are products of active worm activity, suggesting that positivity will be evidence of active infections. In addition, these proteins will minimize the challenges of cross reactivity in coinfections and have a higher specificity compared to antibody response to crude antigen (SEA) in low endemic setting (198, 199). Two gut saposins, SjSAPLP4 and SjSAPLP5, likely to be prominent in *S. japonicum* vomitus have been used in ELISA format to detect schistosome infections in laboratory animals and human patients in China (200) and the Philippines (201). Their orthologs are also prominent in proteomic analyses of *S. mansoni* vomitus. However, like the other antibody-based tests, a positive antibody test to purified schistosome gut proteins may not be a surrogate for worm burden particularly given the difference in gut activity between female and male worms. For instance, the female worm consumes about eight times more blood than the male, presumably producing more vomitus. Nevertheless, it should be possible to improve on the sensitivity of detecting antibodies to these parasite proteins to very light infections, ultimately targeting a single worm pair.

**Figure 1:**
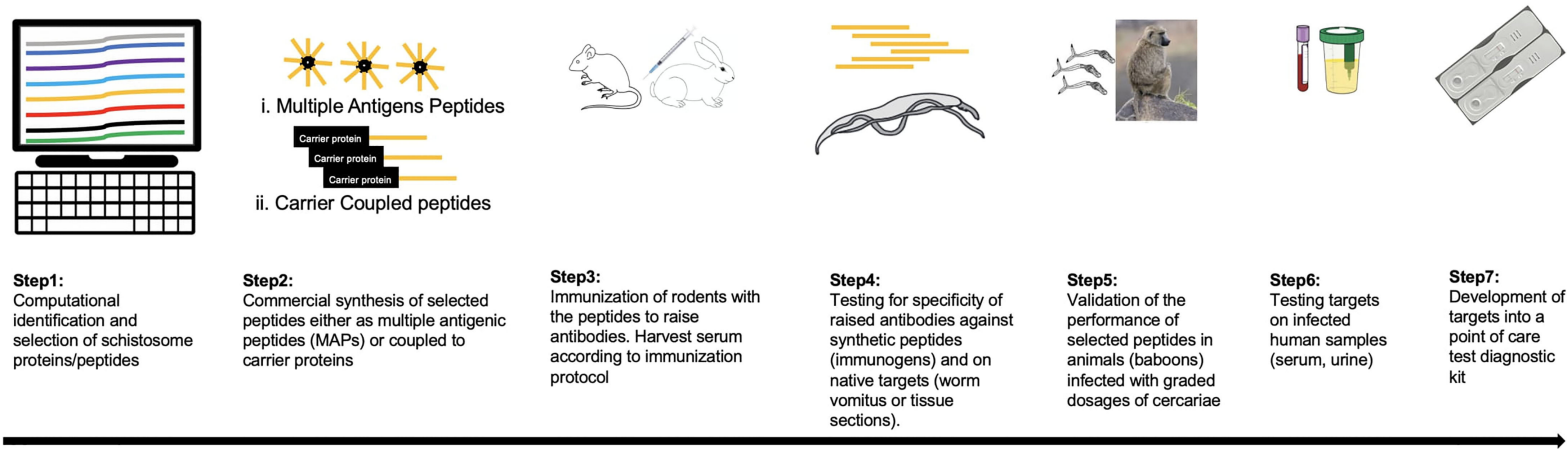
A framework for the development of new diagnostic tagrets for schistosomiasis. Schistosome protein sequences are investigated to identify those that are present in host circulation. By computational approaches suitable peptides are selected based on prediction algorithms (step 1), the best hit candidates (depicted as orange in the figure) are commericially synthesised either as multiple antigenic peptides (MAPs) or conjugated to a carrier protein like ovalbumin (step 2). In step 3, mice/rats/rabbits are immunized with the synthetic peptides to raise antibodies according to the desired protocol. Serum samples are collected serially including pre-vaccination, during vaccination timepoints and after the final dose. In step 4, antibodies in serum can be tested directly (polyclonal antibodies) or can be enriched for a preferred isotype, first against the synthetic peptide or native target like worm vomitus for proteins from the schistomose gut in an ELISA format. For surface proteins, the nataive target can be schistosome tissue sections by immunohistochemistry. Step 5: Promising candidates are validated for sensitivity in larger animals (preferably the baboon model) where graded infections are possible to determine the limit of detection by comparing reactivity of serum to recovered worm burden. Step 6: Candidates are tested against serum and/or urine samples from individuals with confirmed schistosome infection. Finally, additional development steps are carried out to design the validated targets as rapid point-of-care diagnostic kits.

Advancements in Proteomics and Bioinformatics approaches have generated tools that allow identification and selection of peptides of schistosome proteins for production as synthetic peptides (**Figure 1**). These peptides can then be used to immunize rodents to raise polyclonal serum (that can be purified to specific isotypes) and be tested for reactivity in a stepwise manner, first to the synthetic targets and then to their native targets of worm vomitus or parasite tissues. For example, these approaches have been used to demonstrate Cathepsin B as suitable target for immunodiagnosis by antibody ELISA (202–204). It is possible to capture this enzyme in the circulation of infected individuals using a specific polyclonal rabbit antibody (205). Other schistosome proteins identified using similar approaches and detected in infected humans include the tegument surface glycoprotein Sm200 (206, 207). In addition, epitope mapping of exposed tegument and alimentary tract proteins identified several targets recognized by immune sera following vaccination with radiation attenuated cercariae (208).

Secreted schistosome proteins are suitable diagnostic targets, and like the glycan epitopes, have a potential to be designed into point of care rapid diagnostic targets. However, a lot of research is needed to move the lead candidates like Sm31 from ELISA based assays to the strip formats while pursuing novel protein diagnostic targets. To move the sensitivity of these tests to the desired single worm pair detection will require validation using graded infections in animal models and the baboon, in our view and experience (107, 168, 209, 210), is the best model in which to do this. Indeed, we have a catalogue of serum from our previous studies on vaccinations (92, 106, 131), coinfection studies (94, 95), and graded infections some of which we used in the validation of UCP-LF CAA kits (168).

In summary, the goal of schistosomiasis elimination will be realized when there are available rapid, sensitive, and easy to use diagnostic methods to identify infected individuals even in low transmission settings. There is great potential in detection of parasite derived materials either as glycans or proteins because they are more specific and have better sensitivity than the parasitological tests. Molecular techniques offer improved hope in xenomonitoring, and the LAMP technique has removed the main barrier of expensive machines that is needed for PCR-based assays. Whichever the diagnostic method employed in evaluating the success of control interventions, it is worth considering the possibility of therapeutic failure caused by an incomplete cure due to the sub-curative effect of praziquantel when used at usual doses (128).

## Vaccines Against Schistosomiasis

The WHO advocates for continuous research and development of new tools and treatment approaches for elimination of schistosomiasis. Vaccination is a mainstay for the control of many infections. However, for schistosomiasis, many decades of vaccine research have been frustratingly less fruitful with many promising preclinical candidates failing to reach human clinical trials. Nonetheless, the search for a schistosomiasis vaccine is still warranted as an additional kit to the toolbox of schistosome control strategies. Indeed, at *Science’s* request, 50 experts ranked a schistosomiasis-vaccine as one of the top 10 vaccines requiring urgent development based on feasibility and need (211). The clamour for a schistosomiasis vaccine is made even more urgent by the fact that despite praziquantel being effective against all species of schistosomes, schistosomiasis prevalence has remained largely unaffected, first because of reinfection in high transmission areas and second due to maturation of juvenile worms that were not cleared by the drug, both of which restore the prevailing levels of infection after each round of MDA programme (212). A point of concern is that widespread use of praziquantel would likely lead to development of drug resistance in the future. Since schistosomes do not multiply within the human host (and other vertebrate hosts of importance), a schistosomiasis vaccine does not need to be 100% effective, and mathematical modelling supports that even a partially protective vaccine would contribute to reducing schistosome infections and interrupting transmission (213–215). Considering the schistosome lifecycle, the chink in the armour of the worms that would be most desirable for targeting with a vaccine in humans is the short interval between cercarial skin penetration and the presence of schistosomula in the lungs when the parasite should be the most vulnerable for immune attack as it adapts to the definitive host (212). Targeting this early stage of the parasite in humans is also appealing because there are no complications of immunopathology that is often associated with the eggs deposited by the adult worms.

The first human vaccine trial was against *S. haematobium* (rSh28GST, aka Bilhvax) (216) which ended with rather disappointing results because sufficient efficacy was not reached. Three other vaccine candidates - Sm-TSP-2 (*S. mansoni* tetraspanin 2), Sm-p80 (*S.mansoni* calpain), and Sm-14 are in early phase 1/2 trials (217, 218) and preliminary results have not been made public yet. The status of schistosome vaccines and vaccine development have been previously reviewed extensively in (212, 219–222). The transmission of *S. japonicum* is complicated by the zoonotic nature of the disease with water buffalos and cattle which are the major reservoir hosts and are responsible for nearly 90% of environmental contamination of parasite eggs (223). For the case of *S. japonicum* therefore, vaccination of bovines is deemed necessary in a strategy dubbed ‘transmission blocking vaccination’ because it would assist in long term prevention of human (and animal) infection (212, 214, 223, 224).

The radiation-attenuated (RA) cercariae vaccine consistently confers protection against schistosomiasis across several species of experimental animal models (92, 225–228) but for practical and ethical reasons, the RA vaccine cannot be used in humans just yet. However, advancements in systems biology (229) and catalogues of highly immune sera from previous animal vaccination studies provides an opportunity to identify very immunogenic proteins from *schistosomes* elicited by the RA vaccine. Indeed, sera from *S. mansoni* RA vaccinated mice and IFN-γ receptor knockout mice were recently used to identify peptides from over 40 proteins that can be prioritized for investigation in multi epitope vaccine constructs (208).

The recent developments in the field of vaccinology for other ancient diseases - malaria and tuberculosis (230–233) - can invigorate efforts to keep refining efforts to look for a vaccine against schistosomiasis. In addition, the availability of novel adjuvants that can selectively manipulate the immune responses and improvements in immunological research makes it possible to assess the specific responses each vaccine needs to elicit through cell signalling studies (234). Perhaps the observation in rhesus macaques where infected animals self-cure (235–237) is an opportunity to understand how the host immune response to successful infection overcomes parasite immune evasion to attain self-cure. This will provide insights into how the vaccines could be engineered to achieve better efficacy. It can be envisaged that the efficacy of such vaccines would benefit most from better diagnostic tools as opposed to the widely used parasitological tests. Ultimately, as we have highlighted throughout this review, and by others (98, 212), the successful control of schistosomiasis will rely on a combination of strategies to achieve meaningful progress and a vaccine against schistosomes is one of those tools that would fill in the gap left by the short-term benefits of MDA programs.

## Potential Benefits of Reduced Prevalence of Helminths on Immunity and Vaccination Against Other Diseases

Chronic parasitic helminth infections are characterized by induction of T helper type 2 (Th2) lymphocytes that produce a Th2 cytokine profile and polarizes the immune system towards a systemic Th2 bias (23, 238–240). Animal studies using knockout transgenic mice have shown that this polarization of Th2 by helminth parasites is orchestrated by the cytokines IL-4 and IL-13 *via* the IL-4 receptor alpha (IL-4Rα) signaling system (241, 242). The IL-4 *via* the IL-4Rα signaling system also influences the expression of transcription factor Foxp3 in regulatory T cells during *S. mansoni* infection (242, 243) including the trans-differentiation of Foxp3+ Treg into Th2 and Th17 cells (244). Treg cells play a central role in regulation of the immune system by maintaining self-tolerance and in suppressing excessive immune responses that are harmful to the host (245). The overall effect of this is the induction of a Th1/Th2 balance that is skewed towards a Th2 bias that is characterized by generalized immune modulation displaying as hypo-responsiveness to bystander antigens. Naturally, Th1 and Th2 responses display reciprocal antagonism (239, 246) and therefore a strong Th2 environment tends to diminish Th1 cytokine responses, indicating the potential of ongoing helminthic infections to suppress the induction of Th1 immune responses. Strong and polyfunctional Th1 responses are crucial in conferring protective immune responses against several infections including TB, malaria, HIV-1 and SARS-CoV2 (247–250). In addition, some Th1 cytokines such as IL-2 are important for induction and maintenance of plasma cells and production of neutralizing antibodies (251–253). Conceivably, therefore, chronic infections with parasitic helminths have the potential to exacerbate the pathological consequences of certain infections, facilitate secondary infections and suppress vaccine-specific immune responses resulting in dampening of vaccine immunogenicity and protective potential. Thus, a significant reduction of the parasitic helminthic worms as proposed by the NTDs 2021-2030 SDGs would be expected to have indirect benefits towards the control of such diseases as discussed below.

### (i) Chronic Schistosomiasis and Comorbidities

Helminthic worm infections are common in areas where major tropical diseases such as TB, malaria and HIV are also prevalent. In such areas, concomitant infections are the norm rather than the alternative. This section will discuss how chronic infections influence morbidity to three major diseases namely, tuberculosis, malaria and HIV. Available information in humans and animal studies suggest that schistosomiasis and *Mycobacterium tuberculosis* (Mtb) co-infections results in increased morbidity and mortality. By using a pulmonary mouse model of Mtb infection, Monin et al, provides insight into the mechanism by which helminth infections may increase disease severity (254). They were able to show that concomitant *S. mansoni* infection with Mtb resulted in impaired IFN-γ but increased expression of IL-4 producing CD4+ T cells that coincided with increase in lung Mtb burden. They were also able to demonstrate increased formation of type 2 granuloma formation in the lungs with high expression of aginase-1-expressing macrophages that were associated with exacerbated inflammation, leading to increased susceptibility, disease progression and disease severity to TB. Treatment with the anti-helminthic praziquantel was shown to reverse disease severity (254). Human studies looking at gene ontology pathway analysis in children with active *S. hematobium* and *Ascaris lumbricoides* infections identified inhibition of IFN-γ signaling, cellular proliferation and the Th1 pathway by DNA hypermethylation of the Th1 pathway and hypomethylation of IL-4 pathway leading to increased IL-4 production by CD4+ T cells (255).

Malaria infection is associated with a predominant Th1 milieu which if not regulated may lead to systemic inflammation and adverse outcomes (256). Downregulation of the pro inflammatory environment by production of IL-10, IL-27 and TGF-β have been implicated in protection against severe malaria (257, 258). Previous studies have shown that concomitant schistosome and malaria infections may alter disease outcome in an age dependent manner. Studies in co-infected children demonstrated increased severity to infection that was possibly attributed to overproduction of circulating INF-γ (259, 260). In contrast, our studies and others have shown a protective effect during co-infection (94, 261, 262), which may be attributed to production of the anti-inflammatory cytokines IL-10 and TGF-β leading to downregulation of INF-γ as previously suggested (259). Despite showing protective attributes during co-infection, recent studies from our lab have indicated impaired IgG and memory T cell responses that may affect naturally acquired immunity and infection intensity. In this study we were able to demonstrate reversed effect after treatment with the anti-helminth praziquantel (95).

Although strong associations between female genital schistosomiasis (FGS) and HIV-1 infection have not been demonstrated clearly, several cross-sectional and case studies (reviewed by (263–265)) have strongly suggested FGS as a risk factor for HIV-1 acquisition, transmission and susceptibility. The presence of *S. haematobium* eggs in genital tissue has been associated with increased vascularization (266) and the accumulation of CD4+ lymphocytes and macrophages (267), making this a potential contributor to HIV-1 vulnerability. Also, FGS has been associated with a higher frequency of systemic CD4 T-cells expressing the chemokine receptor CCR5 (268) resulting in increased HIV-1 target cell population. Furthermore, cervicovaginal immune activation has also been observed in women with FGS (269). Conceivably, FGS lesions may compromise cervicovaginal immune barriers and possibly result in increased vulnerability to HIV-1 infection. Bearing the above in mind, it is plausible that promotion of diagnostic and treatment programmes as proposed by Hotez et al. (270) and others (264) would confer major benefits towards the reduction of this neglected gynaecological disease of poverty as well as HIV-1 infections especially in the sub-Saharan Africa.

### (ii) Chronic Schistosomiasis and Secondary Infections

Chronic schistosomiasis has been associated with urogenital, hepatic and colorectal cancers and has been attributed to deposition of eggs in the liver and other organs. The sequestered eggs stimulate severe local inflammatory reactions with cellular infiltration that lead to formation of mucosal ulcers, microabscesses and granulomas. Prolonged stimulation by the eggs eventually lead to fibrosis, mucosal hyperplasia, polyposis and pseudopolyposis. Postulated mechanisms involved in schistosome induced cancer include: i) immunomodulation by downregulation of immune surveillance and anti-tumor immunity, leading to accelerated tumor growth. Immunosuppressive cells indicated in this process include myeloid-derived suppressor cells, Th2 natural killer T cells, regulatory T cells, and tumor-associated macrophages (271), ii) induction of oestrogen –DNA adduct mediated pathway, with hydroxylation of oestrogen to form semiquinones and quinones that are major carcinogenic metabolites. These compounds have been shown to react with DNA to form depurinating adducts that downstream form mutations in proto-oncogens and/or tumor suppressors leading to formation of cancer cells and iii) prolonged inflammatory responses and activated complement system may lead to increased DNA mutation rates, particularly of the TP53 allele. Mutations of TP53 has been associated with the reduction of MDM2 (transcriptional target of p53) leading to increased transcription and protein phosphorylation of tumor suppressor p53 (272). Activation of p53 is associated with cell cycle arrest to restore genetic integrity, or induce apoptosis, senescence or ferroptosis to eliminate damaged cells. Mutated p53 proteins on the other hand have been associated with a) increased levels of genetic instability such as interchromosomal translocation and aneuploidy, b) reduced sensitivity to anti-growth signals during malignant transformation of a normal cell leading to accelerated and unchecked cell proliferation, c) enhanced glucose uptake and glycolipids rates to facilitate survival of the fast growing malignant cells and d) promote metastasis (273).

Impaired cognitive development had been described in schistosomiasis infection. Earlier studies categorized cognitive development into four; memory (short and long-term), reaction time, learning and intelligence tests. A systematic review and meta-analysis that included 36 studies of 12,920 children showed that schistosome infection was associated with worse performance in memory, learning and intelligent tests compared to the uninfected or treated (274). In recent years, growing evidence suggests that systemic inflammation may be a major causative factor of neuroinflammantion. Studies using the murine model indicated that systemic infection with *S. mansoni* was associated with increased circulatory proinflammatory cytokines IFN-γ, TNF-α, and MCP-1 and IL-12 (275). Although this group did not examine cytokine levels in the cerebrospinal fluid (CSF), previous studies using an LPS model of systemic inflammation, indicated increased TNF-α and IL-1β in both serum and CSF, but not in brain tissue (275). They speculated increased inflammatory responses in the CSF and brain since observed phenotypic changes in astrocytes and microglial cells only occur due to homeostatic disturbances in the central nervous system. Observations at the prefrontal cortex of infected mice described i) activation of astrocyte and microglia as well as morphological changes often observed in brain trauma ii) expression of oxidative stress-induced transcription factor Nrf2, oxidative damage, iii) Tau phosphorylation that is associated with early degenerative modification of the cytoskeleton (276) and iv) amyloid-β peptide accumulation, associated with neurodegeneration and formation of senile plaques in late stage Alzheimer’s disease (277). The Morris water maze test for cognitive function was performed with results indicative of impaired performance in infected animals, characterized by decreased capacity of learning and spatial memory acquisition. Combination treatment of praziquantel and antioxidants (*N*-acetylcysteine plus deferoxamine) prevented this associated morbidity.

Schistosome infections have been associated with stunted growth suggesting a negative effect on bone homeostasis. Bones form part of the immunologic system by interactions in the bone marrow and play a critical role in body support, control of mineral metabolism and hematopoiesis. The bone and the immune system interact with each other through shared regulatory molecules including cytokines, chemokines, receptors, and transcription factors (278). Bones constantly and in a tightly regulated manner destroy and reform to maintain bone volume and calcium levels. Osteoblasts (OB) and osteoclasts (OC) are cells responsible for bone formation and resorption respectively. The differentiation of OC precursors to OC’s is influenced by the osteoclastogenic cytokine receptor activator of nuclear factor-κB ligand (RANKL) (279). High levels of RANKL has been associated with increased osteoclasts with enhanced resorbing leading to bone loss and destruction as seen in HIV and bacteria-induced periodontitis infections (280, 281). Many cell types are capable of producing RANKL with T and B-cells playing a pivotal role during certain infections (281–283). Using a mouse model of schistosmiasis, Li et al, demonstrated that chronic infection with *S. japonicum* resulted in osteoclast-mediated bone loss. This loss was linked to increased RANKL levels triggered by B and CD4+ T cells (particularly Tfh cells) in the peripheral lymphoid tissues (284). These findings point out the risk of bone loss during infection and highlight the potential benefits of supplementing anti-schistosome treatment with bone therapy.

### (iii) Chronic Schistosomiasis and Vaccine Immunity

There is an increasing body of evidence from preclinical and clinical studies documenting suppression of vaccine responses by concurrent helminthiasis (285–287). Two recent studies that were conducted in Africa found that *S. mansoni* infections lowered anti-measles antibodies in previously vaccinated school children (288), while causing a more rapid decline over time in antibody levels to hepatitis B and tetanus toxoid vaccines (289). Poor immunogenicity to TB vaccination has also been associated with helminths (285). Candidate HIV vaccines have been shown to be similarly affected. For example, *S. mansoni* infection in mice has been shown to down-regulate HIV-1-specific immune responses, resulting in delayed clearance of vaccinia virus in the liver, spleen and lungs (290). Similarly, *S. mansoni* -infected mice had a poor IFN-γ response to a candidate HIV-1 DNA vaccine compared to the uninfected group and elimination of helminth infection restored vaccine-specific IFN-γ responses (291, 292). In our own study, we recently demonstrated that chronic *S. mansoni* infection suppresses the induction and boosting capacity of our first-generation candidate HIV vaccines, SAAVI DNA-C2 and SAAVI MVA-C, in the mouse (293) and baboon models (our unpublished data). Furthermore, we have also reported that antibodies to human papillomavirus (HPV) in the sera of *S. mansoni* -infected baboons were lower compared with sera from *S. mansoni*-free animals (294) following immunization with a clinical HPV vaccine.

In humans, immune development in the fetus is greatly described as infection free until birth and transplacental transfer of mother’s antibodies contribute greatly to the child’s immunity during the early stages of life. Recent studies, however, have suggested that this milieu can be altered by antigen exposure *in utero* by maternal infections. Studies have indicated that newborns of mothers infected with helminth infections such as schistosomiasis and filariasis are sensitized with parasitic antigens with a predominant Th2 environment characterized by production of IL-4, IL-5, IgE and IFN-γ (295, 296). These studies have demonstrated that such *in-utero* sensitization establishes immunologic memory that persists into childhood. Maternal schistosomiasis has also been associated with a suppressed immune state characterized by elevation of Treg cells and IL-10. Active suppression by IL-10 and other regulatory mechanisms may lead to decreased responses to important immune factors involved in vaccine efficacy as well as protection from development of severe infection.

Maternal schistosome infection has been implicated with impaired development of protective IgG antibody to *Haemophilus influenzae* b (Hib) and diphtheria toxoid (DT) vaccines and positively correlated with schistosome induced IL-10 levels (297, 298). In addition, reduced IgG levels to measles vaccine (299) and suppression of BCG specific IFN-γ, that are associated with protection (300) has been reported among preschool children. Using a dual IL-4 reporter mouse model, the group by Cortés-Selva et al., demonstrated that babies born to schistosome infected mothers had reduced circulating plasma cells and peripheral lymph node follicular dendritic cells that correlated with long term reduction in production of IL-4 by iNKT cells. Using single-cell RNAseq after vaccination with tetanus and diphtheria, they identified defects in cell cycle, cell proliferation pathways and reduction of Ebf-1 (a key B-cell transcription factor) in majority of follicular B cells that signify long-term defects in antigen-induced cellular immunity (301). Presence of helminth infections further contribute to low prevalence of allergic diseases in endemic areas. A rodent model of schistosomiasis, that investigated the impact of pregnancy on progress from Th1 to Th2 and final regulatory state, observed that offspring from mothers in Th1 and regulatory state were protected against allergic airway inflammation (AAI) (302). In this study, OVA-specific IL-10 was not elevated and thus highlights a minor role of this cytokine in suppressing responses to AAI. Thus, maternal helminth infection, induces differential and distinct alterations at the fetomaternal interface and this in turn determines how progeny respond to allergens (302).

Some studies suggest that these effects may be reduced or avoided by offering antiparasitic therapy to prevent immunomodulation as demonstrated in studies by Noah where they recorded no association between maternal infection and reduced efficacy to childhood vaccines (303). This was unlike previous studies in which they found maternal infection with malaria, hookworms or schistosomiasis were associated with reduced antibody levels of *Streptococcus pneumoniae* in the infants (303). Other studies have shown that children previously infected with *S. mansoni* and treatment with PZQ had higher anti-measles IgG levels post treatment compared to those not treated (299). These studies highlight the importance of control and prevention of parasitic infections during pregnancy.

## Novel Perspectives for Schistosomiasis Research

Improvements in sequencing methods have changed the landscape of biomedical research through developments of new techniques while improving on the existing ones. In this regard, single cell RNA sequencing (scRNA-seq) that is now possible in separate platforms (304–306) including related technologies that allow profiling of transcriptomic signatures together with protein expression like CITE-seq (307–309) as well as chromatin profiling (308, 310) are excellent tools that can be used for profiling and identification of new vaccine candidates and diagnostics targets for schistosomiasis. In addition, it is important to understand the pathways that are employed by schistosomes to downmodulate human responses to other vaccines, regulate immune responses to other coinfections like malaria, as well as immune evasion mechanisms. The WHO roadmap (1, 2) identifies and advocates for the creation of a repository of biological specimens (sera, urine, and stools) for development, validation, and evaluation of new diagnostic targets as an area that will be crucial in elimination of schistosomiasis. Several research groups, including our own, already have archived peripheral blood mononuclear cells (PBMCs) from previous experiments that are ready sources of biological materials to optimize these techniques to answer schistosomiasis specific questions.

While experimental animal models are highly valuable for hypothesis testing and preclinical investigations before applications to human studies, there exists a massive ‘drop-off’ between findings in mouse studies and other high order organisms including humans. In particular, vaccine candidates against schistosomiasis have usually generated very encouraging results in the mouse model but fail to achieve similar protection levels in other experimental animals including the baboon model (131, 311). It is possible that these vaccines regulate pathways that are uniquely found in mice but not in the other higher order animals. ScRNA-seq and systems biology tools can be used to conduct parallel studies in the mice, non-human primates, and human samples to elucidate these differences in response to similar interventions and identify pathways of convergence. Similar approaches have recently been used in the field of tuberculosis research to identify immune correlates of tuberculosis disease and risk in non-human primates, diversity outbred mice and humans (312). Thus, scRNA-seq is a tool now available for addressing novel schistosomiasis research questions from host DNA-methylome to transcriptomics to changes in protein signatures. DNA-methylation studies in the context of RA vaccination would be an ideal entry point in renewed efforts to understand how RA vaccine protection is achieved across species. Despite the value of nonhuman primates as models of disease we note that the strict ethical requirements and restricted use of NHPs limit their use in extensive research. Overall, the elimination of schistosomiasis (and actually for all helminths) can only be achieved using a combination of different measures especially improved diagnosis, vector control, treatment (including vaccination), education (of patients and decision makers) and integration into the local/national health system and monitoring and management of animal reservoirs in *Schistosoma* endemic areas and finally improved understanding of disease pathogenesis.

## Author Contributions

PO and LO made substantial contributions to the conception of the research topic. PO, RKN, GC, and LO undertook literature research, review, drafting and writing and editing of the manuscript. All authors approved the final version of the document.

## Funding

LO is funded by the African Research Network for Neglected Tropical Diseases (ARNTD) small grants program Reference SGPIII/0210/351. GC is supported by research funds from the South African Medical Research Council (SAMRC) *via* the Strategic Health Innovation Partnerships (SHIP) and the South African Department of Science and Innovation (DSI) *via* the National Research Foundation of South African (NRF).

## Conflict of Interest

The authors declare that the research was conducted in the absence of any commercial or financial relationships that could be construed as a potential conflict of interest.

## Publisher’s Note

All claims expressed in this article are solely those of the authors and do not necessarily represent those of their affiliated organizations, or those of the publisher, the editors and the reviewers. Any product that may be evaluated in this article, or claim that may be made by its manufacturer, is not guaranteed or endorsed by the publisher.
